# Tools for the Quantitative Analysis of Sedimentation Boundaries Detected by Fluorescence Optical Analytical Ultracentrifugation

**DOI:** 10.1371/journal.pone.0077245

**Published:** 2013-10-18

**Authors:** Huaying Zhao, Ernesto Casillas, Hari Shroff, George H. Patterson, Peter Schuck

**Affiliations:** 1 Dynamics of Macromolecular Assembly Section, Laboratory of Cellular Imaging and Macromolecular Biophysics, National Institute of Biomedical Imaging and Bioengineering, National Institute of Health, Bethesda, Maryland, United States of America; 2 Section on Biophotonics, National Institute of Biomedical Imaging and Bioengineering, National Institute of Health, Bethesda, Maryland, United States of America; 3 Section on High Resolution Optical Imaging, National Institute of Biomedical Imaging and Bioengineering, National Institute of Health, Bethesda, Maryland, United States of America; German Cancer Research Center, Germany

## Abstract

Fluorescence optical detection in sedimentation velocity analytical ultracentrifugation allows the study of macromolecules at nanomolar concentrations and below. This has significant promise, for example, for the study of systems of high-affinity protein interactions. Here we describe adaptations of the direct boundary modeling analysis approach implemented in the software SEDFIT that were developed to accommodate unique characteristics of the confocal fluorescence detection system. These include spatial gradients of signal intensity due to scanner movements out of the plane of rotation, temporal intensity drifts due to instability of the laser and fluorophores, and masking of the finite excitation and detection cone by the sample holder. In an extensive series of experiments with enhanced green fluorescent protein ranging from low nanomolar to low micromolar concentrations, we show that the experimental data provide sufficient information to determine the parameters required for first-order approximation of the impact of these effects on the recorded data. Systematic deviations of fluorescence optical sedimentation velocity data analyzed using conventional sedimentation models developed for absorbance and interference optics are largely removed after these adaptations, resulting in excellent fits that highlight the high precision of fluorescence sedimentation velocity data, thus allowing a more detailed quantitative interpretation of the signal boundaries that is otherwise not possible for this system.

## Introduction

Through the analysis of the spatial and temporal evolution of macromolecular concentration profiles after the application of a strong gravitational field, sedimentation velocity (SV) analytical ultracentrifugation (AUC) presents a rich source of information on the size, shape, size-distribution, and interactions of macromolecules in free solution [1]. SV is a classical technique of physical biochemistry, but in the last two decades underwent significant development in theory, data analysis, and instrumentation, that led to a wide range of new applications in many fields [2]–[5]. In particular, SV is widely used in the study of reversible protein interactions, and, due to the strongly size-dependent migration and resulting high hydrodynamic resolution, has significant potential in study of the assembly of multi-protein complexes.

Recently a confocal fluorescence detection system (FDS) for AUC, developed by Laue and colleagues [6], [7], has become commercially available, which offers the possibility to monitor the sedimentation of proteins at low nanomolar concentrations or below. In principle, this has the potential to extend the range of protein interactions that can be studied by analytical ultracentrifugation to much higher affinity, such as exhibited in many membrane receptor interactions, antibody-antigen interactions, and interactions in signal transduction. First applications have been reviewed recently by Kingsbury & Laue [8]. Several characteristic deviations of FDS data from the shapes of concentration sedimentation profiles have been described, including sloping sample plateaus [7], [9], dependent on the focal depth of the optics perpendicular to the plane of rotation [7], and the attenuation of the signal close to the bottom of the solution column [7], [9], [10]. Furthermore, imperfect stability of the signal intensity has been suggested to limit the quality of fit of FDS data with standard sedimentation models [11], and the potential of non-linearity in the signal response has been considered [7]–[9], [11], [12].

The goal of the present work was to unravel some of the factors seemingly confounding a quantitative analysis of FDS SV data at higher signal/noise ratio. To this end, we continued on the strategy for AUC analysis of expanding the direct least-squares modeling of signal boundaries with numerical models of sedimentation to mimic experimental conditions. Previous examples of this strategy are models for the finite time of rotor acceleration [13], the finite time of optical scanning [14], and the accommodation of time-invariant and radial-invariant noise offsets into the analysis of interference optical data [15], [16]. Similarly, we have included in the present work temporal and spatial gradients of signal intensity for the analysis of FDS data, as well as a simple model reflecting the geometry of the detection optics. This allowed us to identify and account for the dominant sources of deviations from standard sedimentation models, and to validate the linearity of the detection and precision of the macromolecular sedimentation parameters derived from FDS data over a wide range of sample concentrations and data acquisition conditions.

After accounting for the characteristics of the detection, we consistently obtained fits with excellent quality, with signal/rmsd ratios of fits generally being superior to conventional absorbance detection, and equivalent to that of interference optical SV experiments. We believe that understanding these technical factors will enhance the potential for the application of FDS in quantitative studies of interacting systems.

## Methods

### Fluorescence-Detected Analytical Ultracentrifugation (FDS-AUC)

Analytical ultracentrifugation (AUC) experiments were conducted in an Optima XL-A analytical ultracentrifuge (Beckman Coulter, Indianapolis, IN) equipped with a fluorescence detection system (AVIV Biomedical, Lakewood, NJ).

Enhanced green fluorescent protein (EGFP) was prepared as described previously [12], [17]. A dilution series was made with EGFP dissolved in phosphate buffered saline (Cellgro, Corning; 5.62 mM Na_2_HPO_4_, 1.06 mM KH_2_PO_4_, 154 mM NaCl, pH 7.40) at 14 concentrations ranging from 5.5 nM to 5.56 µM, in the presence of 0.1 mg/ml BSA, all ran side-by-side in 12 mm centerpieces in the same 8 hole rotor.

Prior to the first run the FDS settings were adjusted at a rotor speed of 3,000 rpm, after which the centrifuge was stopped and the samples resuspended. A standard FDS calibration centerpiece was used containing fluorescein according to manufacturer's instructions (10 µM in 10 mM TRIS, 100 mM KCl, pH 7.8). The rotor was then temperature equilibrated to 20°C while resting in the rotor chamber for at least 90 min after the centrifuge console temperature reading showed 20.0°C. This was followed by acceleration to 50,000 rpm. In most experiments, the laser was allowed to warm up for 10–20 min. The PMT was adjusted to a setting of 32%, and angles of data acquisition were verified to be centered and well within each sector. Data were acquired in 0.002 cm radial intervals, and for all gain settings of 1, 2, 4, and 8. The scans were acquired in 1 min intervals, from which data sets with 50–100 scans could be assembled that evenly represented the entire sedimentation process with a total of ∼15,000–20,000 data points. After the SV run, the solutions were gently mixed to resuspend the EGFP. In this fashion, a series of experiments was conducted systematically varying the focal depth, using values (in this order) of 8,000 µm, 989 µm, 3,955 µm, 6,055 µm, 2,055 µm, 3,055 µm, 5,055 µm, 7,055 µm, followed by replicates at 8,000 µm, and 989 µm. This created a total number of 560 data sets, comprehensively covering a wide range of concentrations, and spanning the entire available range of gains and focal depths.

### Data Analysis

Based on the initial analysis of data we created a series of adaptations of the direct boundary Lamm equation modeling capabilities of SEDFIT, accounting for imperfections in the alignment of the fluorescence optics, for temporal drifts of the signal, for shadow effects of the excitation close to the bottom of the cell, for the finite radial resolution, and for signal non-linearity.

The radial signal gradients were modeled in a transformation **T**_rt_ as
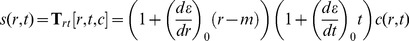
(1)with *r* and *t* denoting distance from the center of rotation and elapsed time since start of the experiment, *m* denoting the meniscus position, *s*(*r,t*) denoting the measured radial and temporal signal evolution, and *c*(*r,t*) denoting the corresponding evolution of macromolecular concentration (in signal units at t = 0 sec at the meniscus) that is simulated by Lamm equation solutions χ(*r,t*) [18]. The symbol *ε* represents a space-and-time-dependent magnification factor, relating the actually measured signal *s* to concentration *c* with *s = ε×c*. The quantity (*dε*/*dr*)_0_ represents first-order approximation from a Taylor series of the radial magnification profile *ε*(*r*), and the term (*dε*/*dt*)_0_ represents the equivalent first-order approximation of the temporal drift *ε*(*t*). Concentration parameters resulting from the analysis are thus in signal units at the meniscus at the start of centrifugation.

The effect on the excitation and emission of the shadow by the centerpiece (and possibly by the aluminum cell assembly) at the bottom of solution column was described as a transformation **T**_S_ as
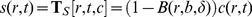
(2)with *b* denoting the bottom (i.e. highest radius) of the solution column and *B*(*r, b, δ*) is the fractional reduction of intensity at a distance *b-r* from the bottom of the cell, given a characteristic diameter 2*δ* of the detection (or excitation) beam. As a first approximation we assumed a geometry where the beam is a cone with opening angle θ that has a circular cross-section in the plane where the beam enters the solution column, and we took for *B*(*r, b, δ*) the fractional area of a circular segment of the beam cross-section formed by the intersection with a straight line at distance *b-r* from the center of the beam. At values *b-r*<*δ*, this leads to
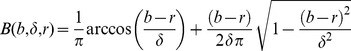
(3)which is a function that increases from 0 at r = b–δ smoothly to 0.5 at r = b. At values r<b–δ, B(r, b, δ) was set to constant 0.

The effect of the finite width of the detection and excitation cone was empirically modeled as
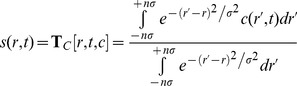
(4)i.e., a radial convolution with a Gaussian with full width at half maximum 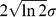 (∼1.67 σ), truncated in each directions to a few multiples of σ for computational efficiency (using *n* = 3 by default). The convolution **T**_C_ is applied after **T**_rt_, and **T**_S_.

Since the transformations **T**_rt_, **T**_C_, and **T**_S_ defined in Eq. 1, 2, and 4 are linear, there can be imposed on each Lamm equation solution in a mixture or in a distribution prior to the distribution analysis [19]. They can be combined as usual with algebraic direct boundary analysis of TI and RI noise contributions to the total signal as described [15], [16].

Finally, due to the potential for inner filter effects producing non-linear signals, an approach to analyze data subject to signal non-linearity following a power law of the form
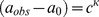
(5a)was implemented. Here *a_obs_* denotes the observed nonlinear signal, *c* the concentration, and *κ* a power coefficient. The offset *a*_0_ was introduced to represent a baseline signal and to restrict the application of the power-law transformation to the macromolecular signal. A theoretical analysis of the impact of such non-linearity on the observed molecular weights, sedimentation coefficients, and diffusion coefficients in sedimentation velocity is presented in **Theory S1**. Unfortunately the non-linear relationship of Eq. 5a precludes the standard distribution analysis that is based on linear combinations of Lamm equation solutions. This problem can be solved, however, by back-transforming the observed data into units that are linear in concentration,
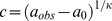
(5a)and performing the distribution analysis in this space. Baseline contributions, such as TI and RI noise parameter can also be calculated in this linearized space using standard methods. The fitted data can then be transformed again into the data space for inspection and evaluation of the residuals. (This forward transformation into the original data space is default in our implementation in SEDFIT, but optionally the data and fit can also be inspected in the concentration space.) The disadvantage of this computational approach is obviously that data errors become distorted from the transformation. However, given the superb signal-to-noise ratio of the FDS data at high fluorophore concentration this should have negligible impact on the analysis, and be outweighed by far from the advantage of this approach of enabling the distribution analysis that accounts for sample imperfections and polydispersity, as well as systematic noise decomposition.

For our experiment series, data were analyzed with the c(s) method [19], allowing for TI noise, the refinement of the signal-average frictional ratio, as well as the meniscus and bottom position. In addition, as described above, we accounted for radial magnification gradients and temporal drifts of the detected intensity (Eq. 1) by refining parameters (*dε*/*dr*)_0_ and (*dε*/*dt*)_0_, for shadowing of the solution column close to the bottom of the cell (Eq. 2) by refining the parameter *δ*, and for radial signal convolution (Eq. 4) by refining the convolution width σ.

In order to handle the large number of data sets that can be generated with the FDS system, serial analysis tools were created in SEDFIT to automatically sort the fluorescence data at different gains, to create list-files for each sector and gain allowing to load data sets with scans at given time span and intervals, and to conduct an automated serial analysis for each run that saves each best-fit configuration, performs integration on the derived distributions, and creates a file with the summary of the results. Optionally all data, fits and residuals as well as corresponding distributions can be automatically passed on to the plotting program GUSSI for inspection and the creation of publication quality graphs.

Software tools for the modeling of fluorescence optical data described above, as well as for serial analysis are part of the SEDFIT software version 14.3 and later, which can be downloaded from https://sedfitsedphat.nibib.nih.gov/software/default.aspx. The tools can be found in the Options menu, or be invoked with the keyboard shortcut Alt-F. All plots of AUC data and *c*(*s*) distributions were created with the software GUSSI, kindly provided by Dr. Chad Brautigam, which can be downloaded from the MBR Software Page (http://biophysics.swmed.edu/MBR/software.html).

## Results

### Focal Scans

**Figure 1:**
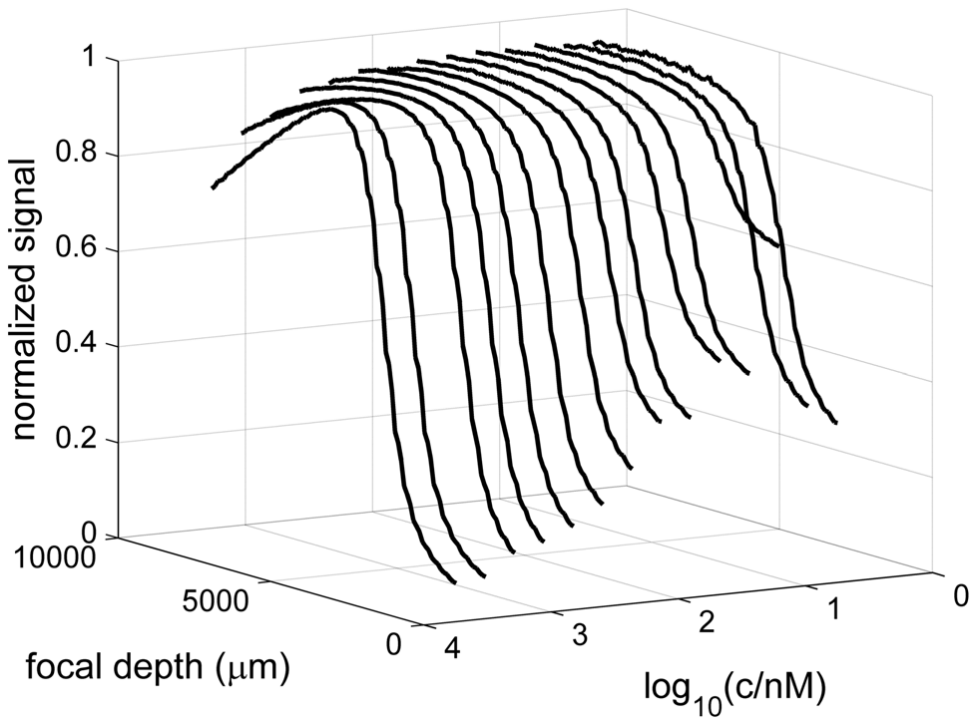
Focal scans in *z*-direction perpendicular to the plane of rotation into EGFP samples at various concentrations, taken radially in the middle of the solution column. The focal scans were normalized (division by the maximum signal in of each focal scan) to better compare their shape at different concentrations.

As reported previously [20], with the FDS system it is not only possible to scan the fluorescence profiles in the radial dimension at different angles (corresponding to different sample sectors), but also conduct scans at constant radius along the *z*-direction moving the focal point of the optics parallel to the axis of rotation to different points inside the sample (for a sketch of the different axes see **Figure S1**). This is not only possible for the calibration cell, but for all sample solution columns, which allows us to study some properties of the optical detection of the samples. A superposition of such focal scans taken radially in the middle of the EGFP sample column at low rotor speed effectively prior to sedimentation is shown in **Figure 1**, normalized relative to the loading concentration of our EGFP samples over the range of 5.5 nM to 5.56 µM. FDS acquisition at small focal distances places much of the sensing volume outside the sample volume, whereas larger focal distances produce maximal signal. Interestingly, for samples above 100 nM, but not for lower sample concentrations, there is a drop in signal for the largest focal distances, creating a peak in the focal scan. The drop at higher concentrations is thought to be due to inner filter effects [11], [20]. Although it is the recommended practice to place the focal point for the SV experiments at the maximum of the focal scan [11], [20], a difficulty that arises is that the maximum is dependent on sample concentration, which initially motivated us to acquire SV data at a range of focal depths to study the impact on the observed SV boundaries.

**Figure 2:**
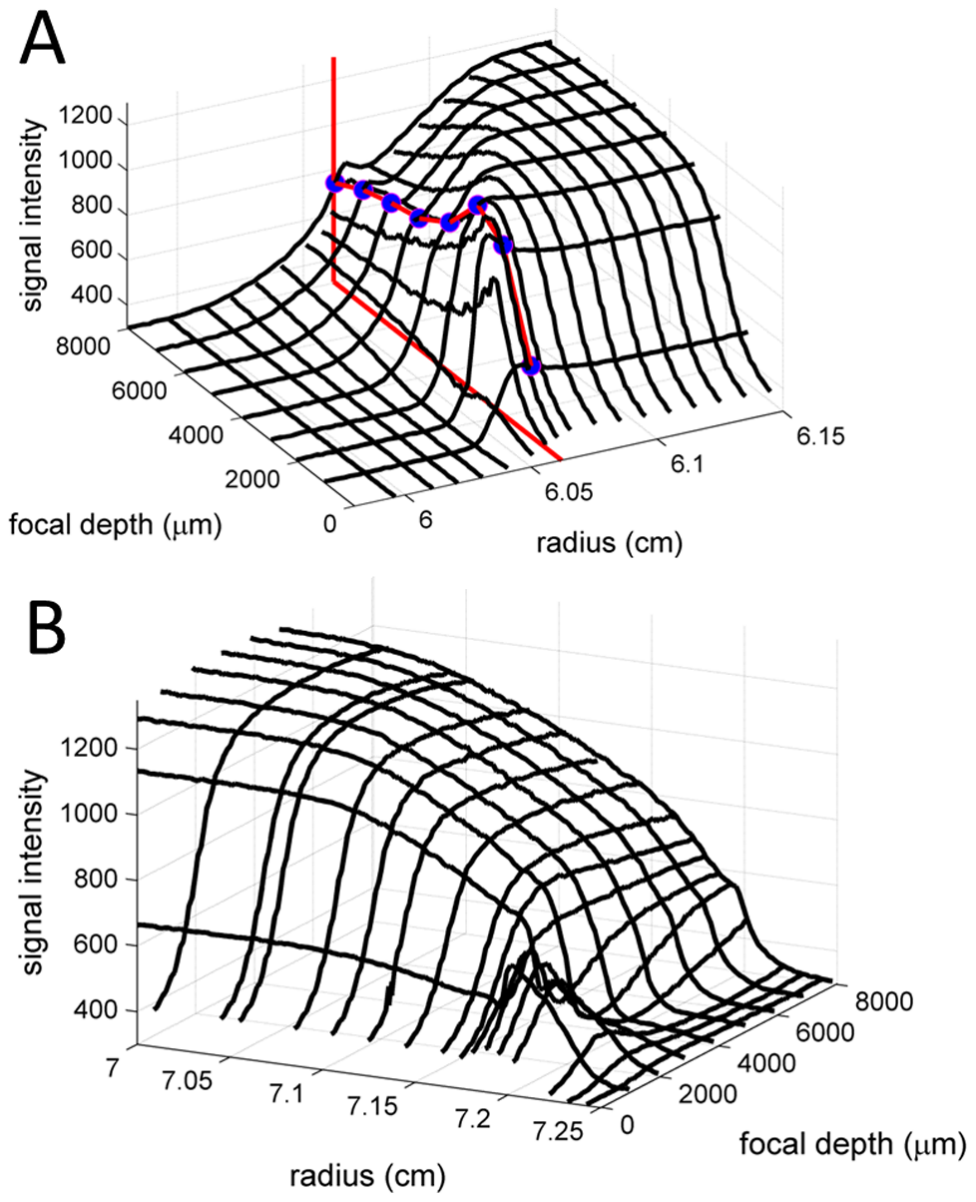
Signal intensity as a function of radius and focal depth in the vicinity of the meniscus (A) and bottom (B). Shown are the signals of EGFP at 100-fit meniscus position from analysis of the high-speed SV experiment is shown after correction for rotor stretch (red lines in the axes planes; measured signals at the meniscus position are shown as blue circles connected with a red line).

Another interesting aspect is the signal obtained as a function of focal depth and radius at either end of the solution column, as it is informative on the geometry of the optical data acquisition. **Figure 2** shows signals at meniscus (lowest radius) and bottom (highest radius), acquired under low speed conditions where the sample concentration is virtually uniform and can be thought of as a step function at the ends of the solution columns. At the meniscus, the signal increases smoothly already outside the solution column and assumes its maximal value well inside the solution column. It can be discerned that the width of this feature increases with increasing focal depth. This increase in apparent width of the transition of the same solution column at the same rotor speed with increasing focal depth is consistent with a finite emission or detection cone causing increasing signal convolution with increasing focal depth. Only a small feature seems to point to the presence of the meniscus, at a position very close to the calculated best-fit meniscus position from a high-speed SV analysis (red lines; for clarity the radial scan values at the meniscus position are highlighted as blue circles). Likewise, the signal smoothly drops towards the end of the solution column at the bottom, steeper at higher focal depths. In contrast to the detection at the meniscus, the signal appears to be truncated.

### Radial Signal Gradients

**Figure 3:**
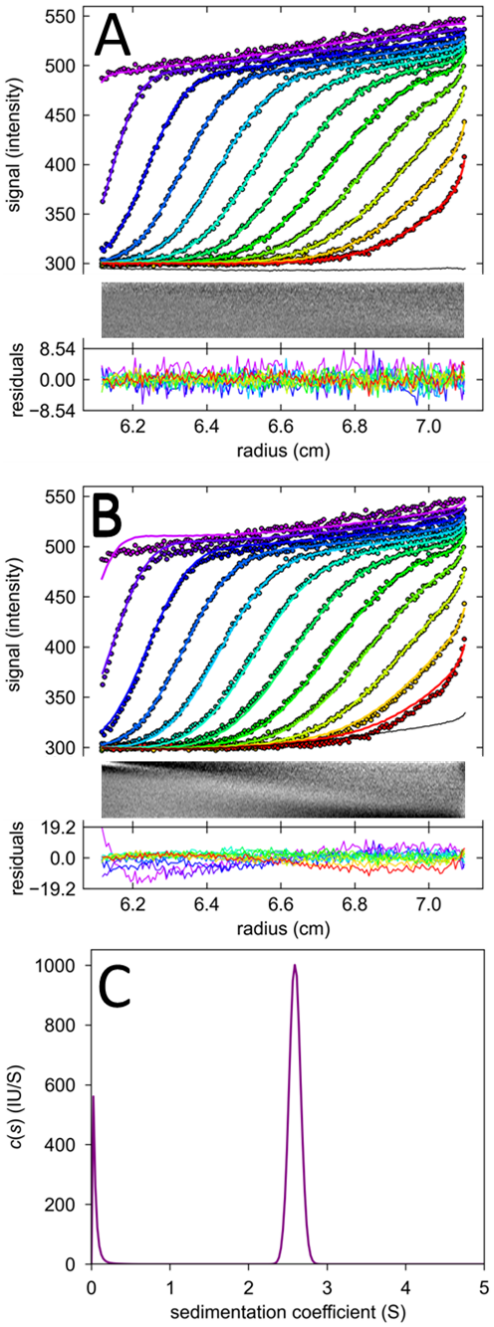
Radial gradients of signal magnification produce sloping solution plateaus. Shown are FDS-SV data of 135 nM EGFP acquired at a focal depth of 989 μm. (A) Experimental scans (symbols) and best-fit model (solid lines) using a model incorporating TI noise and a radial magnification gradient with best-fit value of *dε*/*dr* = 0.28, leading to a best-fit rmsd of 1.79 counts. The TI noise is shown as black line. The sedimentation model is *c*(*s*), resulting in a single peak at 2.59 S with frictional ratio 1.39, corresponding to an apparent molecular weight of 31.9 kDa. Residuals are shown in the lower panels, as residuals bitmap and overlay plot. (B) Analogous representation of the same data modeled without radial magnification gradient, which leads to a curved TI profile partially compensating for the sloping plateau. The rmsd of the best fit is 3.42 counts. The peak s-value of *c*(*s*) in this fit is 2.65 S, with a frictional ratio of 1.67 corresponding to an apparent molecular weight of 43.3 kDa. (C) Sedimentation coefficient distribution *c*(*s*) corresponding to the correct model show in Panel A.

For data sets acquired at small focal depths we consistently observed positive slopes in the solution plateaus but not in the solvent plateaus (**Figure 3**). This behavior has been reported previously by Kroe & Laue [7] and was attributed to imperfect alignment of the FDS relative to the plane of rotation, such that the focal point travels in *z*-direction while scanning radially. Since changes in the focal depth can change the magnitude of the observed signal (**Figure 1**), especially at low focal depths (or large focal depths at high concentrations), the imperfections in the alignment will lead to changes in the magnification of the signal across a radial scan.

Although the use of TI noise to compensate for this effect was proposed previously [7], we found that this did not give good fits to the data (**Figure 3B**). Rather, considering that a mechanical mis-alignment seems well-described by a constant function *dz*/*dr* in radius, and because the ensuing changes in focal point are thought to be small compared to the curvature *dε*/*dz* of the focal scans, a first-order correction of intensity of the radial signal (*dε*/*dr* = *dε*/*dz*×*dz*/*dr*≈*const*) from the small changes in the localization of the focal volume appears to be reasonable. This is computationally easily achieved by introducing a single parameter, as described in Eq. 1, and one additional parameter is commensurate with the additional information content provided by the data in the form of the clearly discernible slopes.

We tested this approach with experimental data collected in the FDS. In order to maximize the information content of the experimental data of sedimentation velocity with any optical system, it is important (and common practice) to collect scans that evenly represent the entire sedimentation process, from the start of initial depletion near the meniscus until after the disappearance of the trailing edge of the boundary near the bottom.

Throughout, this approximate correction was found to fit this feature of the data extremely well, as shown, for example, in **Figure 3A**. In addition to providing a better fit, the model with the radial gradient of signal intensity magnification led to a single *c*(*s*) peak (**Figure 3C**). Thus, what from superficial visual inspection of a few scans based on the experience, for example, of data from the absorbance optical system, may be mistaken for the signature of a polydisperse sample, turns out to be a single species, which can be discerned from the c(s) plot of **Figure 3C**. Quantitatively polydispersity can be clearly distinguished from radial magnification gradients by virtue of including scans representative of the entire sedimentation process. Furthermore, the best-fit frictional ratio of the *c*(*s*) fit results in a value of 1.39 for EGFP, corresponding to a reasonable apparent molecular weight of 31.7 kDa. This is close to the expected molecular weight of 31.1 kDa based on the amino acid composition of the His6-tagged protein. By contrast, the attempt to model this data with only TI noise compensating for the sloping data converges at a best-fit frictional ratio of 1.67, corresponding to a clearly overestimated molecular weight of 43.3 kDa.

**Figure 4:**
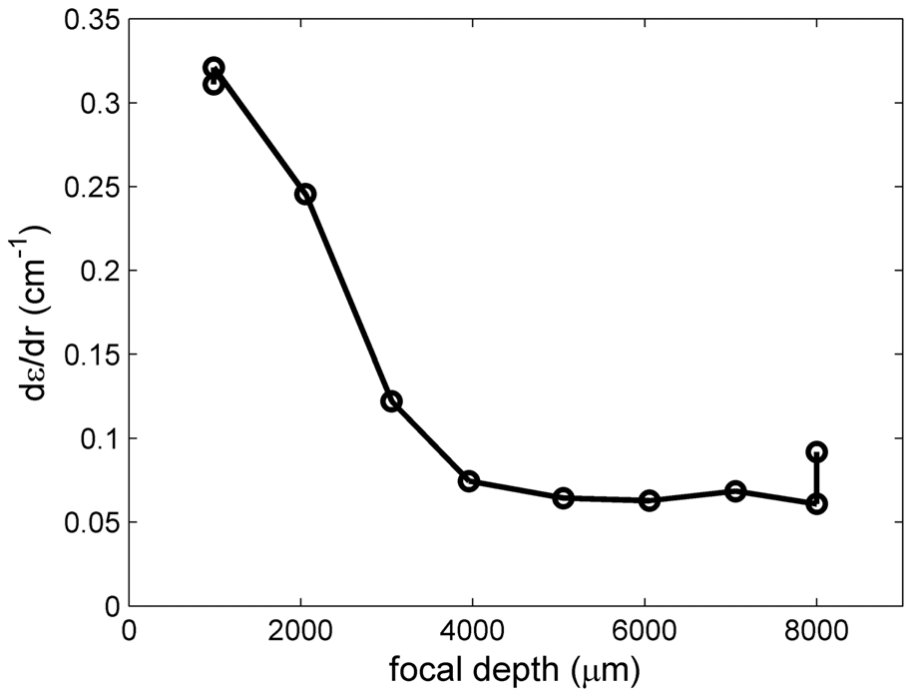
Dependence of the radial magnification gradient on the focal depth. Shown are the best-fit values of dε/dr for a sample of 27.4 nM EGFP.

The highest best-fit values for *dε*/*dr* were obtained at the lowest focal depths, decreasing to insignificant contributions at focal depths >3000 μm (**Figure 4**). This is consistent with the expectation, considering that the steepest slopes of the focal scans *dε*/*dz* are observed in the range of focal depths below 4000 μm. Considering the slopes of the focal scans *dε*/*dz*, the observed values of *dε*/*dr* ∼ 0.2–0.3 cm^−1^ suggest misalignment of the radial rail out of the plane of the rotation, *dz*/*dr*, on the order of a few hundred μm per cm, or an angle on the order of one or a few degrees. Very similar observations were made across the entire concentration range studied, with similar values at all concentrations (data not shown). The focal depth dependence appears inconsistent in the present data with a second, alternative origin of such errors described by Kroe & Laue [7], where systematic imperfections in the timing of scans relative to the angular rotation of the sample lead to radial-dependent temporal truncation of the signal intensity.

### Temporal Signal Drifts

**Figure 5:**
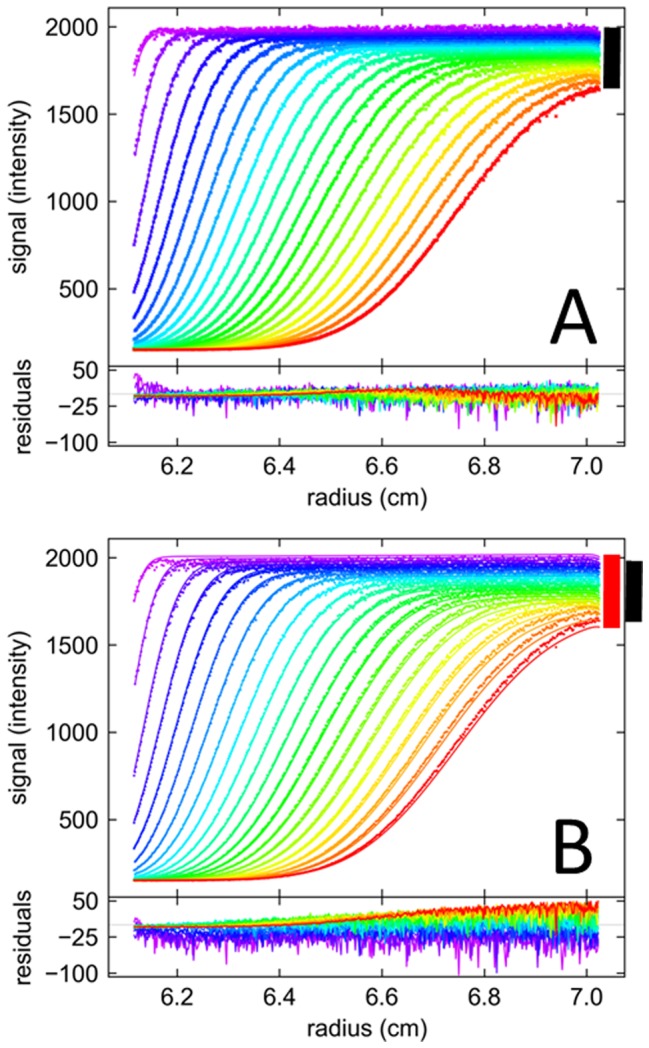
Illustration of the temporal magnification changes. FDS-SV data of EGFP at a concentration of 664 nM were acquired at a focal point 3955 μm. (A) The data (crosses) were fitted with a single species model (lines) incorporating a radial gradient of signal magnification *dε*/*dr* with a best-fit value of 0.0079 cm^−1^, and a temporal drift *dε*/*dt* of 0.0127 h^−1^. (B) Based on the best-fit model of Panel A, boundary profiles were calculated for the same data with identical model parameters but eliminating the temporal drift, setting *dε*/*dt* = 0. No further fit was done in Panel B, except an adjustment of the macromolecular concentration parameter. In order to highlight the difference in the boundary shapes, the black bar reflects the measured radial dilution (A), whereas the red bar reflects the radial dilution in the absence of signal drifts based purely on geometry of sedimentation (B). The residuals reflect the difference between the data and the model without temporal drift correction.

A second feature that is very apparent on closer inspection of FDS data of sufficient signal is a deviation from the expected decrease of solution plateau with time. An obligate, basic consequence of sedimentation in the radial geometry is an increase in intermolecular distance, or radial dilution. Because radial dilution is directly linked to the migration of the boundary, the observed spacing of the solution plateaus clearly indicates additional processes affecting the signal intensity with time. This is illustrated in **Figure 5**, where the experimentally observed decrease of the plateau signal for a selection of experimental scans is shown as a black bar, which can be compared with the red bar that indicates decrease in plateau signal that would be expected for a sedimenting species with the same sedimentation parameters and normal radial dilution, in the absence of time-dependent intensity changes.

It can also be discerned from **Figure 5** that the time-dependent intensity changes are different from time-dependent baseline offsets (as in RI noise contributions), since the solvent plateau does not exhibit any change with time. Further, we can exclude signal non-linearity from possibly causing this deviation from expected signal levels, as very similar effects were found across the entire range of concentrations used in the present study.

Two obvious sources of time-dependent variation of signal intensity are drifts in the laser power, as well as photo-physical effects, mainly photo-bleaching. We performed an independent experiment under low-speed, non-sedimenting conditions to determine the stability of the detected fluorescence intensity with time, and, over the course of one day, observed initially positive and then negative slow drifts with the order of magnitude of 0.1–1% per hour, consistent with a slight increase in laser power and with photo-bleaching (data not shown).

For modeling experimental FDS-SV data over the shorter time-scale of the EGFP experiment (7 hours), a constant drift (*dε*/*dt*)_0_ appears a reasonable first approximation to account for this instability. Higher-order approximations of *ε*(*t*), for example as quadratic functions, should be possible but do not seem warranted. Throughout all data sets from the 10 independent FDS-SV runs with 14 different EGFP concentrations conducted in the present study, the addition of a single parameter (*dε*/*dt*)_0_ reflecting a temporal intensity drift was suitable and led to virtually perfect fits of this feature of the data. For data at suitably high signal/noise ratio this led to a 2–3 fold improvement of the rmsd.

Across different cells of the same run of our EGFP experiment series, the best-fit values of (*dε*/*dt*)_0_ were typically consistent within 0.5% per hour, and somewhat different in different runs. However, at large focal depths a consistent correlation with sample concentration was observed (**Figure S2**).

### Beam Truncation and Radial Convolution

**Figure 6:**
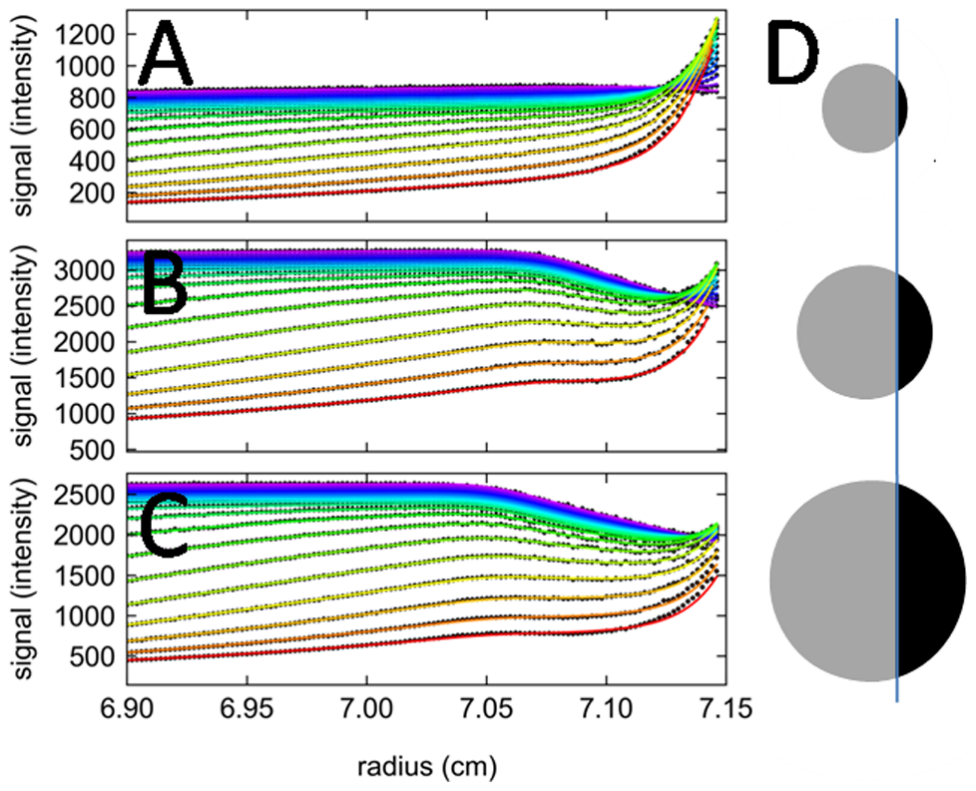
Comparison of the effect of beam truncation close to the bottom of the cell at different focal depths. Shown are the experimental FDS-SV data of 1921 nM EGFP (symbols, only every 2^nd^ scan shown), along with best-fit models based on Eq. 3 and convolution Eq. 4 (lines). Focal depths are 989 μm (A), 5055 μm (B), and 8000 μm (C). The graphics in Panel D illustrates how smaller beam cross-sections (grey), centered at the same radius, are obscured to a different extent by the bottom of the cell (black), thus leading to a partial loss of intensity.

Dependent on the focal depth, FDS-SV data show a characteristic overall drop in signal intensity close to the bottom (highest radius) of the cell (**Figure 6**). We have used a geometric model based on the simplifying assumption of a uniformly illuminated circular beam cross-section (with diameter 2δ) that becomes increasingly obscured by a wall at distances smaller than *δ*, taking the fractional loss in intensity as the fractional remaining visible area (**Figure 6D**). The shadow region will start when the beam center is at the distance from the bottom that equals the beam cross-section radius *δ*, and the shadow will reduce the detected intensity exactly by half if the beam is centered at the bottom radius. The functional form of the intensity loss in this model is given by Eq. 3. As shown in **Figure 6**, it describes the experimentally determined signal profiles very well. Since the decrease in signal and its slope is very characteristic and cannot be confused with back-diffusion – although it can be partially visually obscured by back-diffusion it at later times – the parameter *δ* can be treated as a parameter to be refined in the fit.

**Figure 7:**
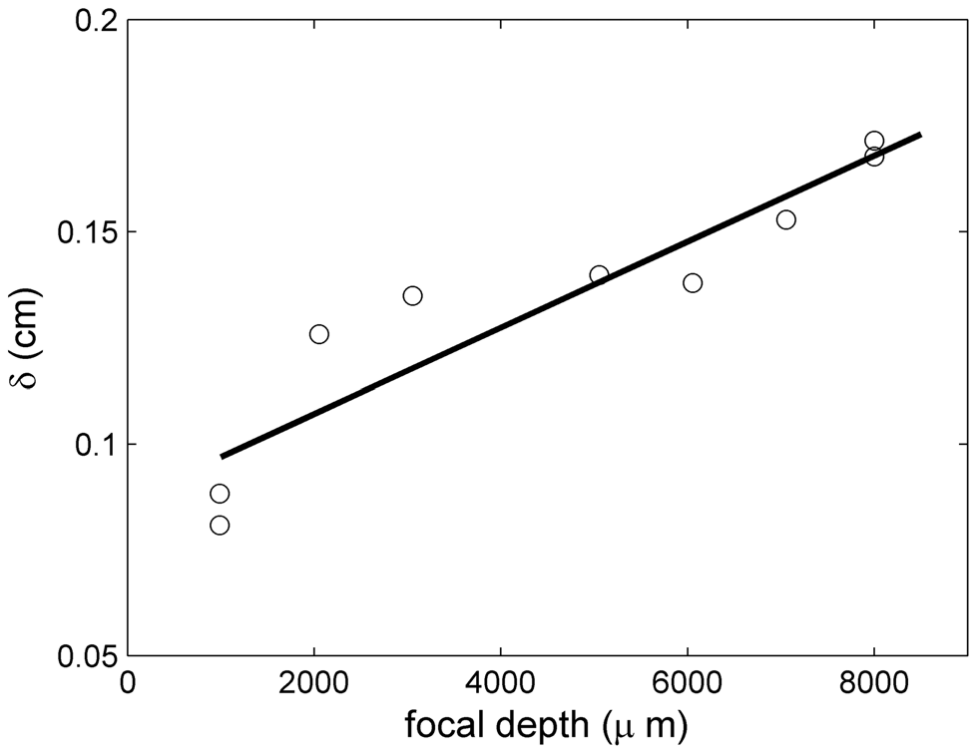
The dependence of the best-fit beam radius *δ* (Eq. 4) on focal depth (circles). The solid line is a linear fit, leading to an apparent beam angle of 5.8°.

The change of best-fit beam diameter with focal depth (**Figure 7**) and the slope *dδ*/*dz* offers an opportunity to calculate an effective beam angle 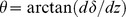, which from the data in **Figure 7** is 5.8°. Based on the numerical aperture of the focal lens the maximal beam angle is 8.21°, but the power density profile of the beam is not known, making these quantities difficult to compare.

Finally, we can expect several factors to impact the radial resolution, including the pin-hole size of 100 μm, the beam divergence and power density. Radial signal convolution can be discerned, for example, at the onset of shadow close to the bottom of the solution column, where a sharp edge would be geometrically predicted by Eq. 3, but a smooth transition is observed. Radial convolution is revealed more clearly at the meniscus, where no physical obstruction of the light path from the sample holder exists. Under conditions of low rotor speed in the absence of significant sedimentation the radial scans should ideally represent a step functions. However, as shown in **Figure 2A**, a broad and smooth transition can be discerned from baseline signal at smaller radii in the air space far from the meniscus to the sample plateau signal far inside the solution column. In theory, the convoluted signal of a step-function will reveal the integral over the convolution function. Thus, motivated by the similarity of the observed transitions with an error function, and lacking more detailed knowledge of the beam geometry, we have included radial convolution *ad hoc* as a Gaussian convolution (Eq. 4). With values of σ converging typically below 0.03 cm, this has only a negligible impact on the boundary model, except for smoothing the edge at the onset of shadow close to the bottom predicted by Eq. 3, not taking the beam power profile into account.

Potentially complicating factors for the interpretation of the signals at the meniscus are reflections and refraction of the excitation and detection cones at the air-water interface. In order to experimentally determine the magnitude of these factors, we conducted FDS experiments of 100 nM EGFP at 3000 rpm with a standard solution column, side-by-side with a solution column overlaid with mineral oil to create an oil/water interface instead of the air/water interface. Since mineral oil has a refractive index slightly above that of water, much closer to water than air to water, different refraction and reflection effects should arise. However, qualitatively very similar patterns were observed in focal and radial scans in the vicinity of the meniscus (**Figure S3**), suggesting that reflection and refraction are not dominant factors.

### Combined Application of Corrections and Analysis of Signal Linearity

The analysis tools outlined above were applied to analyze the complete set of 560 SV data sets of EGFP acquired at 14 different concentrations, 10 focal points, and 4 different gain settings. This was aided using serial analysis tools created in the SEDFIT software in order to process the large number of data sets that can be generated with the FDS system.

**Figure 8:**
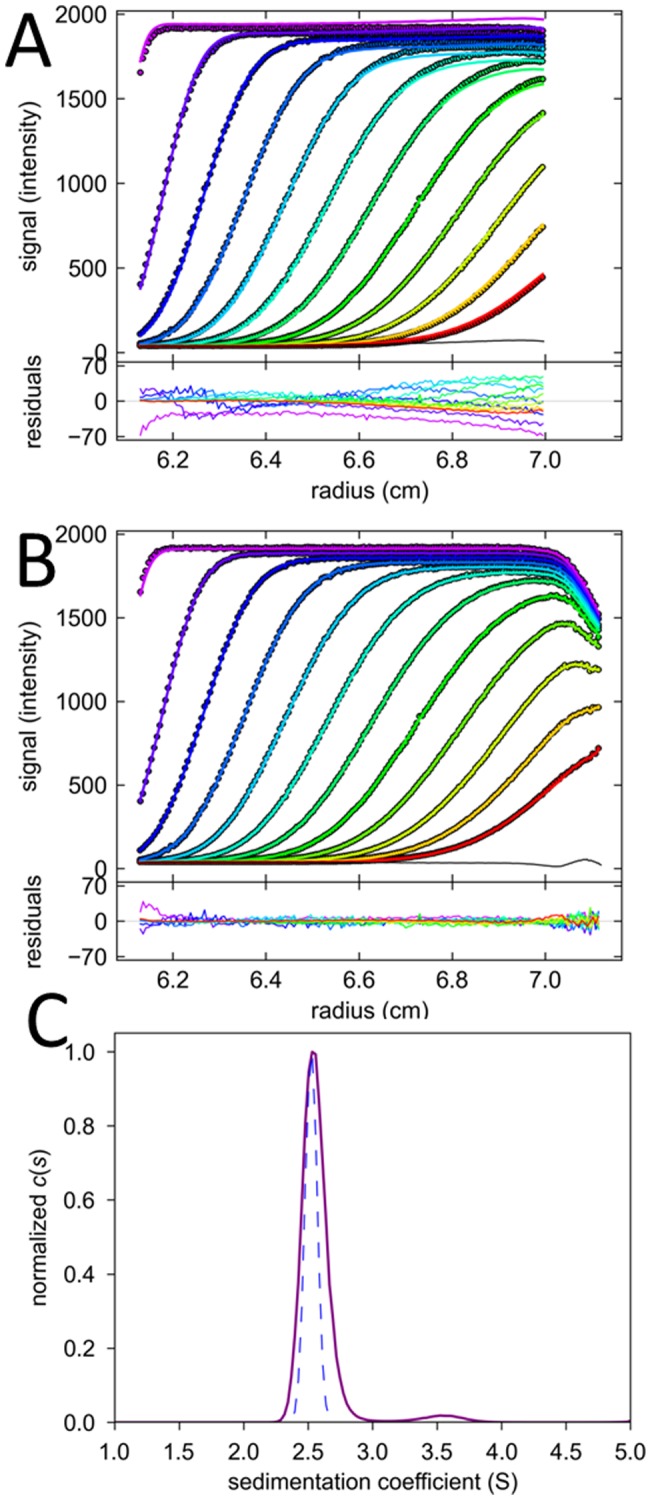
Fit of FDS-SV data at 3258 nM EGFP acquired at a focal depth of 8000 μm. For clarity only 3^rd^ scan and 3^rd^ data point are shown. (A) Fit and residuals of a model without any FDS-specific corrections with standard *c*(*s*), allowing for TI noise (black line). The rmsd of the fit is 17.61 counts. (B) Fit with the same *c*(*s*) sedimentation model but additionally including the corrections for radial magnification gradients with best-fit *dε*/*dr* = 0.0071 cm^−1^, temporal intensity drifts with best-fit *dε*/*dt* of 2.44%/h, a shadow at the bottom of the cell with radius *δ* = 0.183 cm, and a radial convolution of σ = 0.03 cm. The rmsd of this fit is 4.96 counts. (C) Sedimentation coefficient distributions resulting from the fit in (A) shown in blue as dotted line, and from the fit in (B) shown in purple as solid line.

The combination of the corrections described above allow for excellent fits under all conditions, at the lower concentrations resulting in final rmsd values of typically ∼ 0.2–2 counts, or at the higher concentrations values well below 1%, often as small as 0.2–0.3%, of the boundary height. This signal/noise ratio is comparable to highest quality interference optical data. An example at moderate signal and low focal depth is shown in **Figure 3**, another one at high concentration of 3.3 µM EGFP is shown in **Figure 8**. A standard fit is shown in Panel A, leading to large systematic residuals and an unphysical curvature in TI noise. After application of the FDS specific corrections, as shown in Panel B, the rmsd of the fit improves by a factor 3.55 to a final value of 0.26% of the boundary amplitude, at the same time allowing the extension of the radial range of the data that can be incorporated into the fit. Interestingly, the *c*(*s*) distribution only from the corrected fit, not from the naïve conventional fit, shows the population of EGFP dimer (**Figure 8C**), which at low µM EGFP concentrations should be present due to the dimerization of EGFP with *K_D_* on the order of 100 µM or higher, measured independently by conventional absorbance SV (data not shown).

The fact that we can fit the data well with straightforward optical corrections provides an opportunity to precisely measure the signal boundary heights (*via* integration of *c*(*s*)), measured relative to the meniscus at the start of centrifugation, as a function of loading concentration, and thereby assess signal linearity.

**Figure 9:**
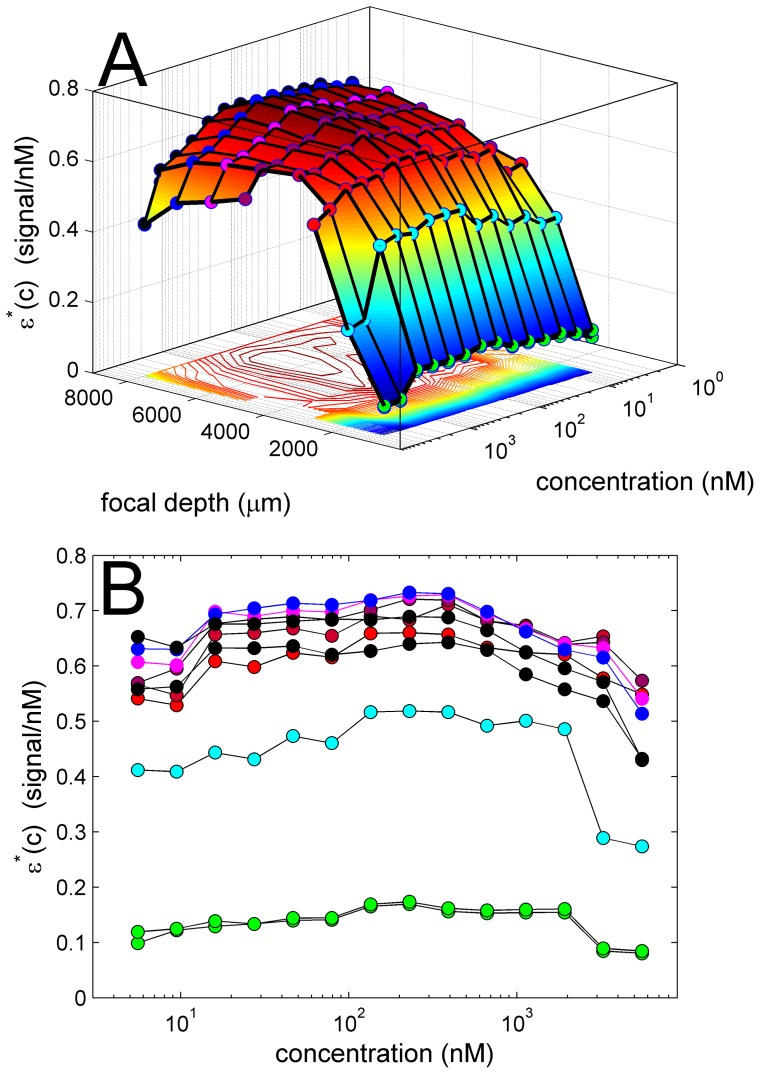
Measured signal increment ε*(*c*) as a function of EGFP concentration and focal depth at a photomultiplier voltage setting of 32%. The observed boundary amplitudes after integration of *c*(*s*) were normalized with regard to a gain setting of 1 and with regard to loading concentration to calculate the specific signal per nM concentration at different conditions. A surface and contour plot (A) and overlay (B) are shown for the same data. For clarity, experimental series with different focal depth are highlighted with markers of different color: 989 µm in green (two sets), 2055 µm in light blue, 3055 µm and 3095 µm in red, and 5055 µm in purple, 6055 µm in magenta, 7055 µm in dark blue, and 8000 µm in black (two sets).

First, for each same sample at concentration *c*, the best-fit signal boundary heights *a_b_*(*c,g*) obtained from data acquired in the same run at different gain settings were compared. Without exception, a near perfect linear relationship *a_b_*(*c,g*) *versus g* was observed with residuals typically less than 1% of the average boundary heights (data not shown), suggesting the signal amplification to be excellent. From the linear fit *a_b_*(*c,g*) *versus g* we determined the slope a_b_*(*c*) = d*a_b_*(*c,g*)/d*g* as an average signal boundary height, which incorporates the information from all gain settings and is normalized relative to a gain of unity. Since the values a_b_*(*c*) span as many orders of magnitude as the concentration range used in the experiments, and log-log plots are notorious for hiding deviations from linear relationships, we divided the signal boundary by the loading concentration, ε*(*c*) = a_b_*(*c*)/*c*, as an effective signal increment in units of signal per nM concentration. This would clearly show non-linearity of the signal, for example, from inner filter effects in the form of a characteristic decrease of ε*(*c*) with concentration. The data obtained are shown in **Figure 9**. Not surprisingly, different focal depths yield different specific signal increments across all concentrations, as can be expected from the shape of the focal scans (**Figure 1**). However, whereas the focal scans in **Figure 1** were simply normalized relative to the maximum value at each concentration, the data in **Figure 9** are net boundary amplitudes normalized by sample concentrations. This allows us to quantitatively compare signals at different sample concentrations. Specifically, it can be discerned that, at any given focal depth, the specific signal decreases slightly at concentrations above ∼500 nM EGFP, a drop that is somewhat exacerbated at large focal depths. (A large drop in signal at the two highest concentrations is also observed for the data at a focal depth of 2055 μm, but since this is in the region of the steepest focal depth dependence of the signal, this may originate from small errors in the focal depth.).

Even though the data in **Figure 9** indicate the presence of some low degree of non-linearity from inner filter effects at high concentrations, on the order of 10–20% per decade, the fit of the 3258 nM EGFP data at a focal depth of 8000 μm could not be significantly improved with a power-law approximation of signal non-linearity (Eq. 5). Adding a power-coefficient to the adjustable parameters we found only a minor improvement of the fit from an rmsd of 4.96 to 4.95 counts, at a best-fit on-linearity coefficient of 1.0036.

### Analysis of Sedimentation Coefficients and Apparent Molecular Weights

**Figure 10:**
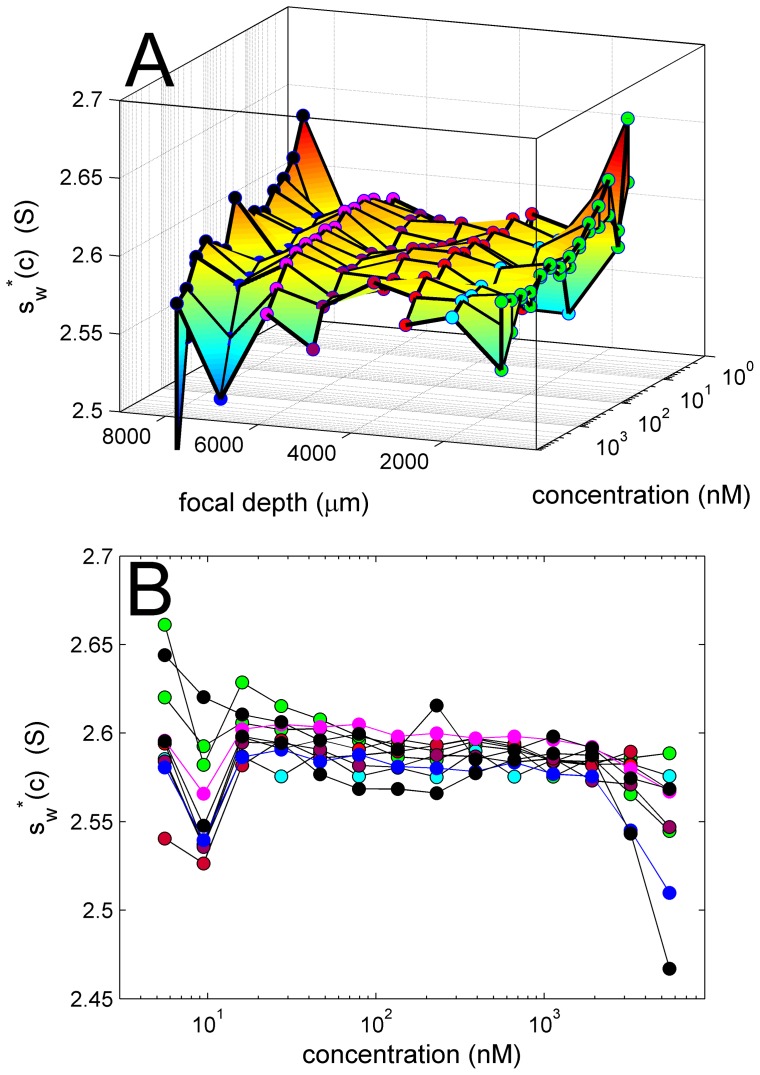
Signal weighted-average sedimentation coefficient s_w_*(*c*) as a function of EGFP concentration and focal depth. s_w_*-values are based on integration of *c*(*s*) and each represent an average of values at different gain setting. A surface and contour plot (A) and overlay (B) are shown for the same data. For clarity, experimental series with different focal depth are highlighted with markers of different color: 989 µm in green (two sets), 2055 µm in light blue, 3055 µm and 3095 µm in red, and 5055 µm in purple, 6055 µm in magenta, 7055 µm in dark blue, and 8000 µm in black (two sets).

Analogous to the linearity of the signal amplitude with gain factors, the weighted-average *s*-values determined by integration of *c*(*s*) was highly consistent for data sets obtained at different gain settings, with an average standard deviation of 0.004 S, or 0.17% of the overall average *s_w_*-value of 2.584 S (uncorrected for buffer density and viscosity). Thus, for the further evaluation we determined the average *s_w_*-value for all gain settings at each concentration and each focal depth, s_w_*(*c*), as plotted in **Figure 10**. First, the values are quite consistent, with an overall relative standard deviation of all values of 0.87%. This is comparable to the accuracy of s-values determined for BSA in conventional absorbance and interference detection an extensive study comprised of many instruments [21]. However, it is somewhat higher that the typically expected repeatability of *s*-values measured in the same instrument [21], but is contributed significantly by the largest scatter at the lowest concentrations where the boundary has low signal/noise ratio. Further, it can be discerned from **Figure 10** that the *s*_w_-values are low at the highest concentration and largest focal depth. This would be consistent with the expected effect of signal non-linearity leading to an increasing lag between the true concentration boundary midpoint and the signal boundary midpoint (**Theory S1**). Finally, it is interesting that there appear systematic errors from run to run, for example, with all s_w_*-values from the run at 6055 µm (magenta in **Figure 10**) being higher at all concentrations relative to those obtained 5055 µm (purple in **Figure 10**). The reason for this is unknown, but could originate from factors other than optical detection.

**Figure 11:**
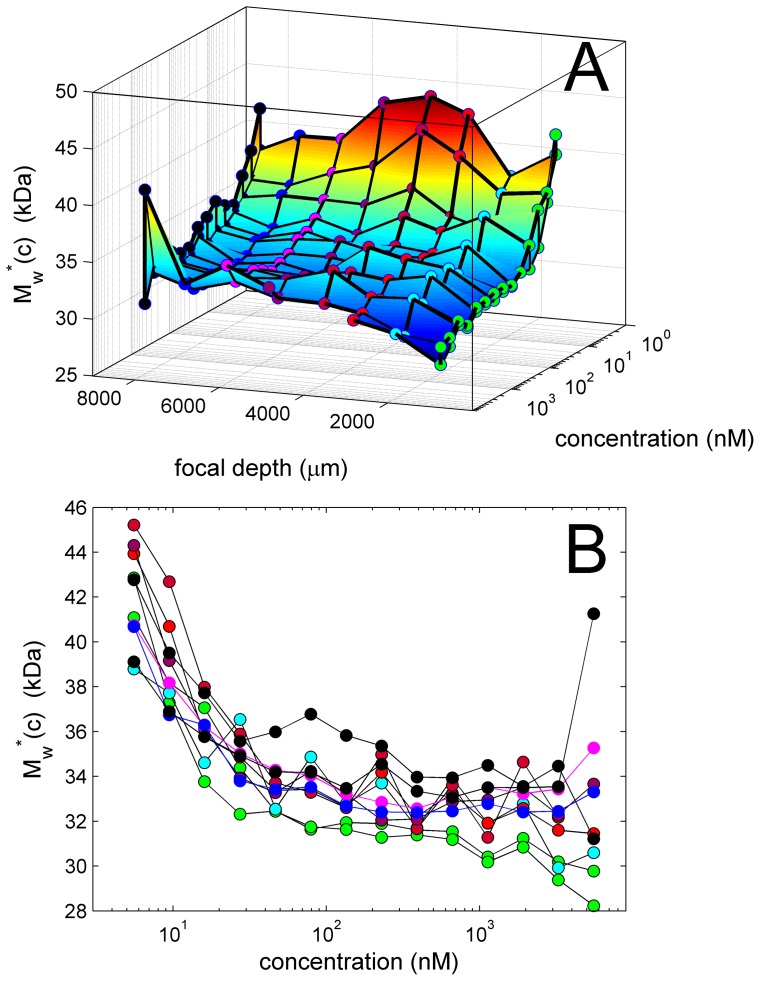
Apparent molecular weights as a function of EGFP concentration and focal depth. M_w_*-values are based on integration of *c*(*s*) and each represent an average of values at different gain setting. A surface and contour plot (A) and overlay (B) are shown for the same data. For clarity, experimental series with different focal depth are highlighted with markers of different color: 989 µm in green (two sets), 2055 µm in light blue, 3055 µm and 3095 µm in red, and 5055 µm in purple, 6055 µm in magenta, 7055 µm in dark blue, and 8000 µm in black (two sets).

Finally, the apparent molecular weight values are shown in **Figure 11**, as implied by the best-fit frictional ratio obtained from modeling the boundary spread. The overall average apparent molecular weight (measured on the basis of a partial-specific volume of 0.73 ml/g) is (34.3±3.1) kDa. As is well-known, the statistical precision of the molecular weight estimates derived from the boundary spread in sedimentation velocity is much less than the statistical precision of sedimentation coefficients. We calculated the statistical error at the lowest three concentrations to be on the order of 10 kDa, which can explain the clear overestimates at low concentrations to be a reflection of poor automated initialization in the analysis. At intermediate and high concentrations the statistical errors are on the order of a few kDa, which is less than the significant scatter observed at the highest concentration. If there was significant non-linearity in the signal, as shown in **Theory S1**, it would lead to a significant underestimate of the molecular weight derived from the boundary migration and spread. The data do not seem to support a decrease in apparent molecular weight with increasing focal depth and concentration. Interestingly, however, small focal depths yield systematically slightly lower estimates, suggesting a larger apparent boundary spread.

## Discussion

In the present work we have introduced tools that adapt the data analysis model in a first approximation to some aspects of experimental data acquisition with the recently introduced commercial fluorescence optical detection system (FDS) [6], [7].

The selection of the depth of the focal point within the solution column is an important experimental parameter when using the FDS [7]–[9], [11]. At too shallow focal points, it becomes most apparent that the line of scanning is not exactly in the plane of rotation, and the ensuing change of focal depth along the radial scan produces a change in signal magnitude [7]. Ideally, therefore, the entire focal volume should be inside the solution column but not so far as to generate inner filter effects reported at large focal depths for high fluorophore concentrations [7]–[9], [11]. Acquiring data in focal scans in the z-direction vertically into the solution column at a fixed radius produces a signal maximum, and it was proposed to place the focal point into the middle of the maximum of the focal scan [11]. This was thought to avoid both inner filter effects at high concentrations and to make the signal insensitive to errors in ‘tracking’. However, a practical problem with this approach is that the position of the maximum is dependent on sample concentration, and that the focal point cannot be changed during the run and is uniform for all cells. Further, in principle, both inner filter and ‘tracking’ effects may still exist at the maximum of a focal scan, which only ensures that they are of equal magnitude initially at loading concentrations.

Thus, instead of achieving a compromise in the experimental design, it seems more desirable to account for ‘tracking’ in the data analysis model. Since it originates from a non-vanishing angle between the plane of rotation and the line of scan, a geometrically motivated linear relationship between radius and changes in the focal depth appears very reasonable. From an analysis of the signal slopes, we estimate the amplitude of the change in z-direction to be on the order of a few hundred micrometers per cm radial movement. Since the focal scans show no strong non-linearity on the scale of a few hundred micrometers, the effects on the signal from ‘tracking’ is that of a simple linear magnification change with radius. The large experimental data set acquired in the present study clearly validate this model (**Figure 3A**), which leads consistently to an excellent fit (in contrast to the previously suggested accommodation of sloping plateaus using TI noise parameters [7], **Figure 3B**). The additional parameter *dε*/*dr* is experimentally well determined, as the slopes in the plateau consist of a very large number of data points. It should not correlate with sedimentation parameters, since even a very broad distribution of sedimenting species that could theoretically mimic a single initial scan with sloping plateau would exhibit a decrease of the magnitude of the slope with time as the larger material sediments. By contrast, magnification gradients stay constant with time. Despite these considerations to make the information content plausible, however, it should be noted that the information on *dε*/*dr* is extracted implicitly from the entire data sets of concentration evolution throughout the entire solution column.

A second optical effect that we have observed in most experiments conducted by us so far, whenever signal/noise ratio was sufficient for a detailed data analysis, is a small temporal signal drift. It can originate, for example, from small changes in the laser power with time, and/or from photo-bleaching. Even though the magnitude of drift does not seem much – in the present experiment series on the order of one percent per hour – due to the highly quantitative nature of SV analysis we found it to have a profound impact on the quality of fit. Signal drifts superimposed to the temporal and spatial evolution of macromolecular concentration distribution seem virtually unavoidable: even if it was possible to perfectly stabilize the intensity of the excitation beam, photo-physical effects in the fluorescent macromolecules appear to be largely out of the control of the experimenter, and will be dependent on the particular fluorophore or on the particular sample. This problem was also discussed by Lyons and co-workers, who observed significant mis-fits of the standard Lamm equation sedimentation model, and discuss the desirability of potential future stabilization of the laser or normalization to a reference sample [11].

Fortunately, we believe this is not essential, because an experimental measure of the overall signal drift is already intrinsic to the sedimentation experiment for sedimentation velocity data with not too broad sedimentation boundaries: The change of the solution plateau signals with time is strictly determined by the sedimentation boundary movement through the geometry of the sector-shaped cell, and thus, the change of the measured solution plateau signals with time and the deviation from the geometric square dilution rule will directly report on the overall signal drift. But even though the information on the drift *dε*/*dt* can be readily visually observed from the compression of the plateau signals, it should be noted that information on the signal magnitude drifts also resides in the entire scans and will be fully exploited implicitly by the direct boundary modeling. In this regard, the temporal drifts are similar to their orthogonal spatial magnification gradients discussed above. In the present work we have implemented only a constant drift parameter, which we found to produce excellent fits. This constitutes a first approximation to a possible more complex time-dependence, which may become necessary to account for, and could be explored in further work. As an additional feature we have also implemented in SEDFIT the option to describe an exponential decay in signal intensity, which may allow the study of fluorophores undergoing more severe bleaching.

A further characteristic feature of all fluorescence optical data, except for those acquired at the smallest focal depths, is an attenuation of the signal close to the bottom of the solution column, caused by components of the cell assembly blocking a part of the excitation and/or detection cone [7]. So far, this required the exclusion of the entire radial range affected by this from the data analysis, amounting to a loss of approximately 1 mm solution column, dependent on focal depth. This requires judgments on the fitting limits to be made, which may be particularly difficult in the presence of smaller macromolecules and under conditions of significant back-diffusion that may partially mask this effect. Even though the use of fluorinated oil has been suggested to create an artificial transparent bottom [7], [10], a different attenuation of signal, perhaps more similar to that close to the meniscus (**Figure 2A**), might be expected even if masked by accumulation of material in the back-diffusion region. Furthermore, the addition of a water/oil interface appears to bear the potential for interactions, at least for some proteins or hydrophobic fluorophores.

It would be desirable to be able to include the masked data in the fitting range, considering that the precision of sedimentation parameters in sedimentation velocity directly depends on the length of the solution observed, and that even the plateau region is informative with regard to spatial and temporal magnification gradients. To this end, we have applied simple geometrical considerations of a partially shadowed cone as a model for signal attenuation, with the cone diameter as a single unknown parameter. In applications to a large number of data sets, it provided consistently a very good description of the signal in that region of the solution column. Furthermore, the dependence of the cone diameter on focal depth is consistent with the numerical aperture of the focal lens. These aspects support the use of the simple model as a first-order approximation. Clearly, it would be desirable to include more detailed optical information, such as the power density distribution of the excitation beam in further work.

In the future, at least in principle, the exact geometry of optical detection could be incorporated into corresponding transformations of the concentration profiles predicted by the Lamm equations prior to modeling the experimental data. This could potentially eliminate the beam diameter as a free parameter, and would also benefit the consideration of convolution, which in the present work was assumed *ad hoc* to be a Gaussian. The attenuation of the signal close to the meniscus, and its dependence on the focal depth, demonstrates the presence of radial convolution of the signal. However, this did not seem to impact as significantly the analysis of the signal profiles as the other parameters discussed above. We did not attempt to model the region close to the meniscus, because light from both excitation and emission cones may be partly subject to reflection and refraction, even if these factors are not the major determinant for the shape of the signals. Correspondingly, the fit of experimental data early in the run usually lacked quality in the vicinity of the meniscus. It seems prudent and well-justified to exclude data from the analysis that is closer to the meniscus than the diameter of the detection cone that is measured from the shadow at the bottom.

The location of the meniscus is important as an indicator of the origin of the sedimentation boundary at the start of sedimentation. In order to better determine experimentally the position of the meniscus, Bailey and colleagues have suggested the use of light oil with a dissolved fluorescent dye [10]. In the present study, however, we did not find this necessary because early scans did allow us to visually recognize the approximate meniscus location, and because the inclusion of scans from the entire sedimentation process usually carries sufficient information for the meniscus position to be determined implicitly in the model of the sedimentation boundary migration. Especially after the additions to the model described above, the signal-to-noise ratio can easily exceed that of absorbance optical data, for which it was shown that the computational approach of determining the meniscus is more precise than the graphical approach [14]. This expectation could be confirmed in the present work from the variation of the best-fit meniscus position in the replicate experiments at different focal depths, which was only 0.0047 cm (data not shown).

In this regard, it is interesting and important to note that the convolution of concentration step at early times at the meniscus produces a shape mimicking an error-function similar to a sedimentation boundary, but with the apparent lagging tail being in the air-to-air space outside the solution column (**Figure 2**). At very low concentrations, which generate data with extremely low signal levels only on the order of the statistical noise, we have previously shown in conventional absorbance optics that the *c*(*s*) analysis can still produce well-determined *s*-values [12]. In such cases, however, the computational method for determining the meniscus will likely not be satisfactory, and an experimental configuration with suspended mineral oil [10], or a separate measurement with the absorbance optics [9], [11] (provided the radial calibration is accurate [21]), may be very useful to constrain the meniscus position. A similar methodology may be appropriate for sedimentation data with extremely broad boundaries. An essential need for the graphical determination of the meniscus determination also arises in the historic data transformation approaches.

As an experimental basis supporting the present work we have collected an extensive series of 560 sedimentation velocity data sets systematically covering the full range of focal depths, amplifier gain settings, and a concentration range spanning three orders of magnitude. After the applications of the tools above, we were able to achieve fits with excellent quality throughout. The ratio of signal to rmsd of the sedimentation model was up to ∼500:1, which rivals or exceeds even the exquisitely precise interference optical data acquisition system. The large number of replicate analyses also allowed us to verify that the new parameters, which we introduced to adapt the model better to the specific optical setup of the FDS, do not lead to increased statistical or systematic errors in the macromolecular sedimentation parameters of interest.

A large fraction of the experimental data, in particular from those experiments collected at low concentrations that led to only low or moderate signal/noise ratios and collected under conditions of medium or large focal depths, could be fitted equally without the FDS specific terms in the model. Obviously, for example, this is the case where the temporal and spatial signal intensity gradients do not yet have an impact exceeding the statistical noise on the data. However, even at moderate concentrations and focal depths significant errors can arise in standard sedimentation models. For example, significant systematic deviations from the best-fit sedimentation model were found by Lyons and co-workers in fluorescence data, but not absorbance data from the same sample [11]. The ability to fit FDS data well not only for the data with lowest signal/noise ratio is critical to have confidence that we fully understand the nature of the signal, and to have confidence in a quantitative interpretation of the best-fit macromolecular sedimentation parameters.

One very important outcome of the present work is that we found little impact of signal non-linearity for the data with general experimental settings in our studies, and that the systematic deviations from standard Lamm equation models seen at higher concentrations are largely explained by spatial and temporal signal intensity gradients. Although there is some decrease of signal above several hundred nM EGFP, and the shape of focal scans suggests the presence of inner filter effects, the remaining non-randomness of residuals (**Figure 8B**) cannot be improved much from applying non-linearity corrections to the model. Further, no significant systematic drop in the *s*-values or apparent molecular weights can be found at high concentrations and focal depths (**Figure 10B**), which would be the expected consequence of non-linear detection, as described in **Theory S1**. Rather, we believe that imperfections in the intensity gradients and optical detection model, intended in the present work as first-order approximations, may be responsible for the remaining small deviations in the present data.

## Supporting Information

Figure S1 **Schematics of the optical and centrifugal setup.**
(TIF)

Figure S2 **Best-fit parameter drift (dε/dt)_0_ obtained for different EGFP concentrations in runs with focal depths of 2055 µm (bold symbols and line) and 7055 µm (thin symbols and line).**
(TIF)

Figure S3 **Radial and focal scans of 100 nM EGFP in the vicinity of the meniscus in the absence (A) and presence (B) of mineral oil layered on top of the aqueous solution column.**
(TIF)

Theory S1 **Theoretical considerations for sedimentation velocity analysis of non-linear signals.**
(DOCX)
