# Supplementary figures and images for: Tools for the Quantitative Analysis of Sedimentation Boundaries Detected by Fluorescence Optical Analytical Ultracentrifugation
Source: PLoS One. 2013 Oct 18;8(10):e77245. doi: 10.1371/journal.pone.0077245 (PMC3799624; doi:10.1371/journal.pone.0077245)

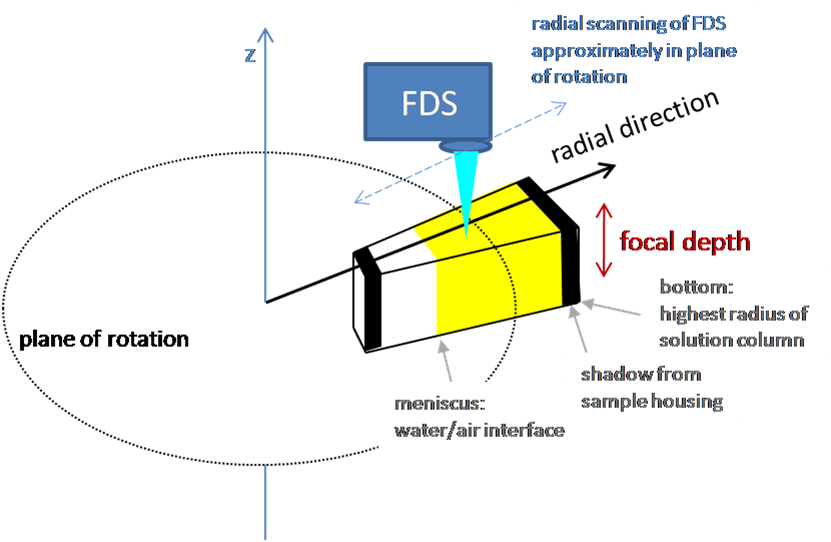

Supplement: Figure S1 — Schematics of the optical and centrifugal setup. (TIF) [file pone.0077245.s001.tif]

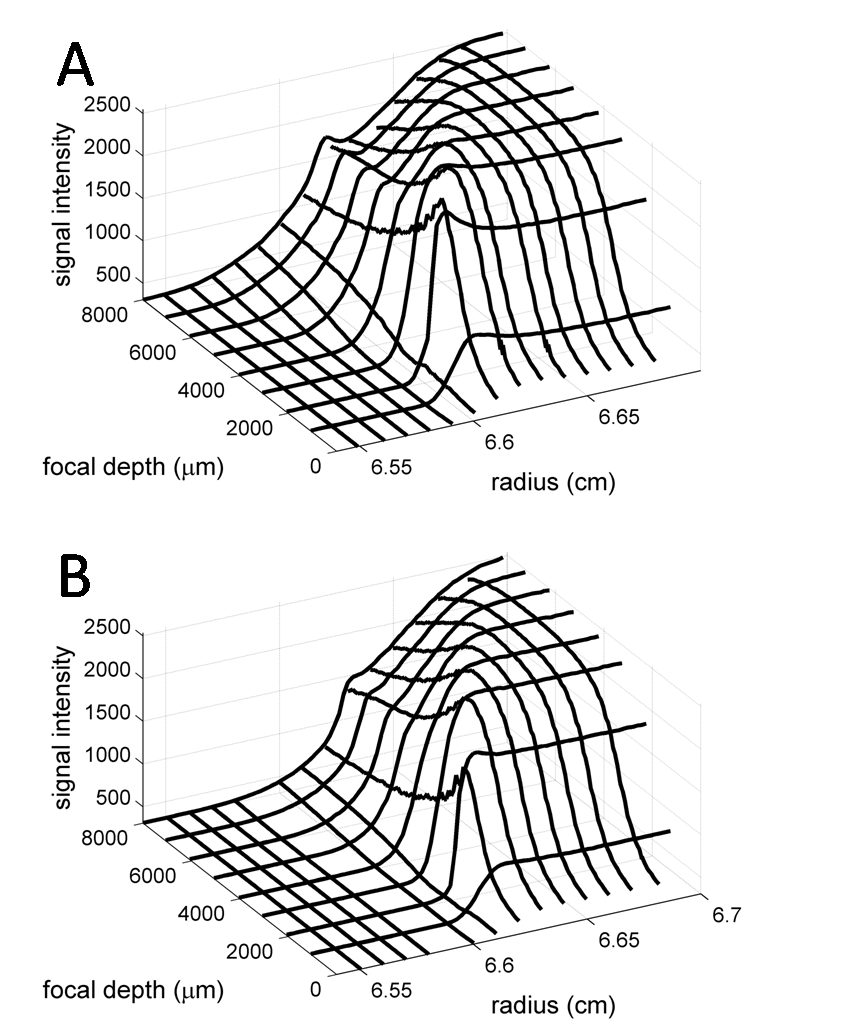

Supplement: Figure S3 — Radial and focal scans of 100 nM EGFP in the vicinity of the meniscus in the absence (A) and presence (B) of mineral oil layered on top of the aqueous solution column. (TIF) [file pone.0077245.s003.tif]
