# Supplementary material for: Tools for the Quantitative Analysis of Sedimentation Boundaries Detected by Fluorescence Optical Analytical Ultracentrifugation
Source: PLoS One. 2013 Oct 18;8(10):e77245. doi: 10.1371/journal.pone.0077245 (PMC3799624; doi:10.1371/journal.pone.0077245)
Supplement: Theory S1 — Theoretical considerations for sedimentation velocity analysis of non-linear signals. (DOCX) [file pone.0077245.s004.docx]

**Tools for the Quantitative Analysis of Sedimentation Boundaries Detected by Fluorescence Optical Analytical Ultracentrifugation**

Huaying Zhao^1^, Ernesto Casillas Jr. ^2^, Hari Shroff^3^, George H. Patterson^2^, Peter Schuck^1^*

^1^Dynamics of Macromolecular Assembly Section, Laboratory of Cellular Imaging and Macromolecular Biophysics, National Institute of Biomedical Imaging and Bioengineering; ^2^Section on Biophotonics, National Institute of Biomedical Imaging and Bioengineering; ^3^Section on High Resolution Optical Imaging, National Institute of Biomedical Imaging and Bioengineering, National Institute of Health, Bethesda, Maryland 20892

**Supporting Information: Theoretical Considerations on Sedimentation Velocity Analysis of Non-Linear Signals**

We assume that the experimental signals as a function of time and radius,$f(r,t)$, can be empirically described – at least over a suitable concentration range – as a function of the local concentration $c(r,t)$ in the form of a power law

$f(r,t)=e^{\alpha+\beta\log(c(r,t))}=A\left(c(r,t)\right)^{\beta}$ (Eq. S1)

. This corresponds to a straight line in a double-logarithmic plot of signal versus concentration,

$\log(f)=\alpha+\beta\log(c)$ (Eq. S2)

, where the slope equals the power law coefficient $\beta$. Obviously, values of $\beta$= 1.0 would indicate a completely linear detection. The power-law approximation of the signal lends itself particularly well to predict the potential effects of signal non-linearity on the sedimentation analysis.

In sedimentation equilibrium, the molar mass can be derived from the slope of the concentration profiles, *M ~ d log(c)/dr^2^*. When this relationship is applied to signals of the form Eq. S1 and Eq. S2, it immediately follows that an apparent molar mass

$M^{*}=\beta M$ (Eq. S3)

will be obtained, i.e., a value reduced in magnitude by the power coefficient $\beta$. For dealing with signal non-linearity one may be tempted to truncate the signal subjected to analysis to a small concentration range where detection is deemed sufficiently linear. However, it should be noted that if the non-linearity of the detection is described by the power-law Eq. S1, then the apparent molar mass would be the same, independent of the concentration range of the data set considered.

In sedimentation velocity, $\beta$-values smaller than 1.0 will lead to the compression of the leading edge of the sedimentation boundary.


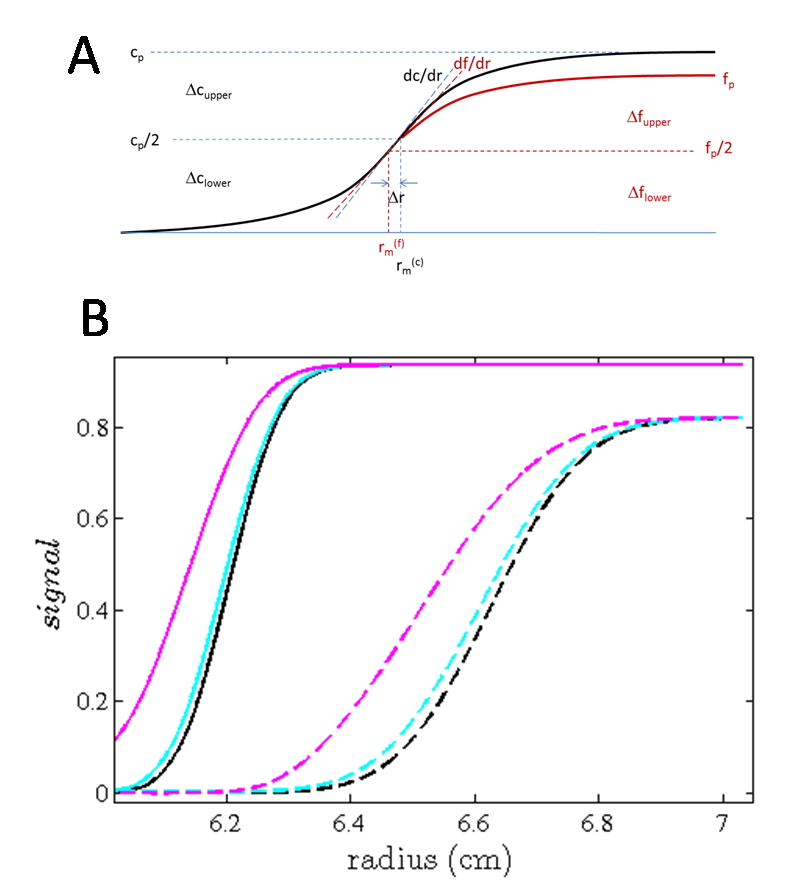


In order to obtain a first intuitive illustration of the effects of potential non-linearity on the sedimentation boundaries of SV, let us consider the boundary midpoint, or more precisely the ‘half-concentration point’ at $c_{p}/2$ (with $c_{p}$designating the plateau concentration at a certain time), and let us calculate the signal ratio of the upper half of the boundary to the lower half of the boundary. In concentration units, of course, they are trivially identical, $\Delta c_{upper}\equiv\left(c_{p}-c_{p}/2\right)=\left(c_{p}/2-0\right)\equiv\Delta c_{lower}$, and $\Delta c_{upper}/\Delta c_{lower}=1$, but in non-linear signal units they would become

$\begin{matrix} \Delta f_{upper}\equiv\left(f(c_{p})-f(c_{p}/2)\right)=Ac_{p}^{\beta}\left(1-2^{-\beta}\right) \\ \Delta f_{lower}\equiv\left(f(c_{p}/2)-0\right)=Ac_{p}^{\beta}2^{-\beta} \end{matrix}$ (Eq. S4)

and lead to the compression factor

$\frac{\Delta f_{upper}}{\Delta f_{lower}}=\frac{1-2^{-\beta}}{2^{-\beta}}=2^{\beta}-1$ (Eq. S5)

. Importantly, we find that for a power-law non-linearity, the compression of the boundary is independent of loading concentration. Quantitatively, for example with $\beta$ = 0.85 the upper half of the concentration boundary would in signal units appear to be only be ~80% the height of the lower half. In signal units, therefore, the ‘half-signal point’ is translated to smaller radii relative to the true ‘half-concentration point’, assuming macromolecular sedimentation – as opposed to flotation – where both the signal and concentration are monotonically increasing with radius.

A time-honored simple strategy for SV data analysis is to follow the displacement of the boundary midpoint with time. We can estimate quantitatively the shift of the apparent boundary midpoint in signal units. Obviously, the magnitude of the radial shift of the signal boundary midpoint, $\Delta r$, relative to the concentration boundary midpoint will be dependent on the slope of the boundary. Since $\Delta f/\Delta r\approx df/dr$, if we denote the boundary midpoint in concentration units as $r_{m}^{(c)}$ and the apparent midpoint in signal unit as $r_{m}^{(f)}$, it follows that the difference in signal at the real and apparent midpoints is $\Delta f=f\left(r_{m}^{(f)}\right)-f\left(r_{m}^{(c)}\right)$, and therefore

$\Delta r\approx\left[f\left(r_{m}^{(f)}\right)-f\left(r_{m}^{(c)}\right)\right]\left(\frac{df}{dr}(r_{m}^{(f)})\right)^{-1}=\left(\frac{df}{dr}\right)^{-1}\frac{f_{p}}{2}\left(1-2^{1-\beta}\right)$ (Eq. S6)

. With Eq. S1, we find the slope in signal units as $df/dr=\beta fc^{-1}dc/dr$. In order to evaluate this derivative at the signal midpoint, we make the approximation of $dc/dr\left(r_{m}^{(f)}\right)\approx dc/dr\left(r_{m}^{(c)}\right)$ since the slope in concentration does not rapidly change (see below), and we linearize the concentration $c\left(r_{m}^{(f)}\right)\approx c\left(r_{m}^{(c)}\right)+\Delta rdc/dr$, which inserted in Eq. S6 leads to

$\Delta r\approx\left[\frac{\left(1-2^{1-\beta}\right)}{\beta-\left(1-2^{1-\beta}\right)}\right]\frac{c_{p}}{2\left(\frac{dc}{dr}\right)}$ (Eq. S7)

. Since the expression in the bracket only depends on the power law coefficient, we introduce the abbreviation $K=\left(1-2^{1-\beta}\right)/\left(\beta-\left(1-2^{1-\beta}\right)\right)$ (where $K<0$ for $\beta<1$), such that

$\Delta r\approx K\frac{c_{p}}{2\left(\frac{dc}{dr}\right)}$ (Eq. S8)

For example, $K$ would be -0.11 for a $\beta$-value of 0.85. $K$can be understood as the fraction of boundary half-width by which the signal midpoint is lagging the true concentration boundary midpoint. Inspection of Eq. S8 shows that the approximation that the concentration slope does not change very much from that of the boundary midpoint is not very good for small$\beta$-values, because they lead to large $\Delta r$, causing an overestimate of the lag. However, corrections for the slope near the boundary midpoint have a zero linear term and start with a term quadratic in $\Delta r$. For evaluating such second-order effects, numerical simulations with Lamm equation solutions were conducted.

For exploring the consequences of the lag, we may use a form of the Faxén approximation of the Lamm equation

$\frac{dc}{dr}\approx\frac{c_{p}}{2}\frac{r_{0}}{r_{m}}\times\frac{1}{\sqrt{\pi Dt}}\times\mathrm{e}^{-\frac{r_{0}^{2}}{4Dt}\frac{(r-r_{m})^{2}}{r_{m}^{2}}}$ (Eq. S9)

with the rotor angular velocity $\omega$, sedimentation coefficient $s$, diffusion coefficient $D$ and elapsed time $t$ after start of sedimentation from the meniscus position at radius $r_{0}$. This provides a simple expression for the approximate slope $dc/dr\left(r_{m}\right)=\left(c_{p}/2\right)\left(r_{0}/r_{m}\right)\left(\pi Dt\right)^{-1/2}$ at the midpoint of the boundary, and leads to

$\Delta r\approx Ke^{\omega^{2}st}\sqrt{\pi Dt}$ (Eq. S10)

. This shows that the lag of the apparent boundary midpoint grows with time, notably independent of loading concentration, but in magnitude dependent on the macromolecular sedimentation and diffusion coefficients. In order to examine the effect of this growing lag on the apparent sedimentation coefficient $s^{*}$ of the signal boundary, we can write the signal boundary midpoint as a function of time

$r_{m}^{(f)}(t)=r_{m}^{(c)}(t)+\Delta r(t)=r_{0}e^{\omega^{2}st}\left(1+K\sqrt{\pi Dtr_{0}^{-2}}\right)$ (Eq. S11)

and apply the usual log transformation of boundary midpoints to estimate the apparent s-value

$s^{*}=\frac{1}{\omega^{2}t}\log\frac{r_{m}^{(f)}(t)}{r_{0}}=s+\frac{1}{\omega^{2}t}\log\left(1+K\sqrt{\pi Dtr_{0}^{-2}}\right)$ (Eq. S12)

or the error in the s-value,

$\Delta s^{*}=s^{*}-s=\frac{1}{\omega^{2}t}\log\left(1+K\sqrt{\pi Dtr_{0}^{-2}}\right)$ (Eq. S13)

. Continuing to approximate the magnitude of the potential effects ensuing from signal non-linearity, but assuming $K$ to be not too large, we use the first term for the Taylor series of the logarithm$\log(1+x)=x-x^{2}/2+...$, which leads to

$\Delta s^{*}\approx K\frac{1}{\omega^{2}r_{0}t}\sqrt{\pi Dt}$ (Eq. S14)

. Obviously, the shift of the apparent s-value is dependent on the time of sedimentation, and as a consequence, in principle, we cannot expect a perfect fit to the signal boundaries, nor the migration of the boundary midpoint. However, towards an approximate and representative expression we may consider an intermediate time when the boundary has reached the middle of the cell, which happens at the time $\tau=\left(\omega^{2}s\right)^{-1}\log\left((r_{1}+r_{0})/(2r_{0})\right)$ if we denote the bottom of the solution column as $r_{1}$. In a typical sedimentation velocity experiment the solution columns extends from $r_{0}$ = 6.0 cm to $r_{1}$ = 7.2 cm, such that $\tau=0.095/(\omega^{2}s)$. Insertion in Eq. 14 leads to

$\Delta s^{*}\approx5.7K\frac{s}{\omega r_{0}}\sqrt{Ds^{-1}}$ (Eq. S15)

, and using the Svedberg equation $D/s=RT\left(M(1-\bar{v}\rho)\right)^{-1}$we can express the fractional error in the s-values of the signal boundaries as

$\frac{\Delta s^{*}}{s}\approx5.7K\frac{1}{\omega r_{0}}\sqrt{\frac{RT}{M(1-\bar{v}\rho)}}$ (Eq. S16)

. For typical parameters of proteins with $\bar{v}$ of 0.73 ml/g, studied in aqueous solutions in SV experiments at 20˚C and at 50,000 rpm, this finally leads to

$\frac{\Delta s^{*}}{s}\approx K\times55\times\left(M/[g\times mol^{-1}]\right)^{-1/2}$ (Eq. S17)

For example, for a molecule with mass of 50 kg/mol, one would obtain a relative error in $s$ of -3% with a $\beta$-values of 0.85.

We may also estimate the error in the apparent diffusion coefficient of the signal boundaries. For simplicity of the analytical calculation, we assume that the slope at the boundary midpoint was used to report the diffusion coefficient $D^{*}$with the Faxén approximation. Applying Eq. S9 at the boundary midpoint in signal and concentration units, we obtain

$\frac{df}{dr}\left(r_{m}^{(f)}\right)/\left(\frac{f_{p}}{2}\right)=\frac{r_{0}}{r_{m}\sqrt{\pi D^{*}t}}$, and $\frac{dc}{dr}/\left(\frac{c_{p}}{2}\right)=\frac{r_{0}}{r_{m}\sqrt{\pi Dt}}$ (Eq. S18)

. In order to evaluate the slope at the signal boundary midpoint, we can apply the chain rule

$\frac{df}{dr}\left(r_{m}^{(f)}\right)=\beta\frac{f(r_{m}^{(f)})}{c(r_{m}^{(f)})}\frac{dc}{dr}\left(r_{m}^{(f)}\right)$ (Eq. S19)

and use the linear expansion for the concentration and its derivative

$\frac{df}{dr}\left(r_{m}^{(f)}\right)/f(r_{m}^{(f)})=\beta\frac{1}{c(r_{m}^{(c)})+\Delta r\frac{dc}{dr}(r_{m}^{(c)})}\frac{dc}{dr}\left(r_{m}^{(c)}\right)\left\{1-\frac{r_{0}^{2}}{4Dtr_{m}^{2}}\Delta r^{2}\right\}$ (Eq. S20)

(where the terms in curly brackets are based on a Taylor expansion of Eq. S9), or,

$\frac{\frac{df}{dr}\left(r_{m}^{(f)}\right)/f(r_{m}^{(f)})}{\left[\frac{dc}{dr}\left(r_{m}^{(c)}\right)/c(r_{m}^{(c)})\right]}=\beta\frac{1}{1+\Delta r\frac{1}{c(r_{m}^{(c)})}\frac{dc}{dr}(r_{m}^{(c)})}\left\{1-\frac{r_{0}^{2}}{4Dtr_{m}^{2}}\Delta r^{2}\right\}$ (Eq. S21)

. After inserting Eq. S8 and using Eq. S18 for the left-hand side, and dropping the higher order terms in $\Delta r$, we arrive at

$\frac{D^{*}}{D}\approx\left(\frac{1+K}{\beta}\right)^{2}$ (Eq. S22)

. With a $\beta$-value of 0.85 this would lead to an approximate over-estimate of the diffusion coefficient by 9.6%, respectively.

Finally, Eqs. S17 and S22 allows us also to roughly estimate the effect on the apparent molar mass estimate from the migration and slope of the boundary midpoint,

$\frac{M^{*}}{M}=\frac{s^{*}/D^{*}}{s/D}\approx\frac{\beta^{2}}{(1+K)^{2}}\left(1+K\frac{55}{\sqrt{M/[g\times mol^{-1}]}}\right)$ (Eq. S23)

. Since *s* is underestimated and *D* is overestimated, the effect is compounded for the determination of the molar mass, although the effect on the diffusion coefficient is dominant. For example, with $\beta$-values of 0.85 for molecules with molar mass of 27 kg/mol, 50 kg/mol, or 100 kg/mol, Eq. S23 predicts underestimates by 12.2%, 11.1%, and 10.5%.
